# Cycles of violence in England and Wales: the contribution of childhood abuse to risk of violence revictimisation in adulthood

**DOI:** 10.1186/s12916-020-01788-3

**Published:** 2020-11-16

**Authors:** Nadia Butler, Zara Quigg, Mark A. Bellis

**Affiliations:** 1 Public Health Institute, Liverpool John Moores University, 3rd Floor Exchange Station, Tithebarn Street, Liverpool, L2 2QP UK; 2 College of Health and Behavioural Sciences, Bangor University, Bangor, UK; 3 Policy and International Health Directorate, Public Health Wales, Clwydian House, Wrexham, UK

**Keywords:** Child abuse, Intimate partner violence, Sexual violence, Physical assault, Revictimisation

## Abstract

**Background:**

Interpersonal violence is a leading cause of death and disability globally, has immediate and long-term impacts on individuals’ health and wellbeing, and impacts global health care expenditures and national economies. A public health approach to violence prevention is crucial, and addressing risk factors is a key priority. Global research has demonstrated that childhood adversity increases risk of a range of poor outcomes across the lifecourse. This study examined the association between being a victim of child abuse and the risk of physical assault (PA), intimate partner violence (IPV), and sexual violence (SV) victimisation in adulthood.

**Methods:**

Data from a nationally representative survey of household residents (adults aged 16 to 59 years; *n* = 21,845) was analysed. Types of child abuse examined included physical, sexual, and psychological abuse and witnessing domestic violence. Logistic regressions examined the independent relationships between child abuse types, experiencing multiple types, and adulthood violence outcomes.

**Results:**

Most individual types of child abuse were significantly associated with each adulthood violence outcome, after controlling for sociodemographics and other abuse types. Compared to individuals who experienced no abuse in childhood, those who experienced one form of abuse were over twice as likely to experience PA in the past year and three times as likely to have experienced IPV and/or SV since age 16 years, whilst individuals who experienced multiple types were three, six, and seven times more likely to experience PA, IPV, and SV, respectively. After controlling for sociodemographics and multi-type childhood victimisation, the type or combination of types which remained significant differed by violence outcome; child psychological and physical abuse were significantly associated with IPV; psychological and sexual abuse with SV; and psychological abuse with PA.

**Conclusions:**

Prevention of child abuse is an important goal, and evidence from the current study suggests such efforts will have a downstream effect on preventing interpersonal violence across the lifecourse. With adulthood victimisation likely to compound the already detrimental effects of childhood abuse, and given that many associated outcomes also represent adversities for the next generation, breaking the cycle of violence should be a public health priority.

## Background

Interpersonal violence is a major public health issue, claiming over 1.3 million lives globally each year [1], and by 2030, homicide is expected to be amongst the top 20 causes of death [2]. Globally, the homicide rate for male populations is approximately four times higher than that for females; however, females comprise over four fifths of homicides carried out by intimate partners and bear the largest burden of victimisation in the context of intimate partner violence [3]. Non-fatal forms of violence are even more prevalent than homicide and have serious consequences for lifelong health and wellbeing [4]. Globally, 35% of women have experienced either physical and/or sexual intimate partner violence or non-partner sexual violence in their lifetime [5]. Recent evidence estimating the health burden associated with intimate partner and sexual violence shows that women who experience either form of violence are approximately twice as likely to have alcohol use disorders and more than twice as likely to have depression, compared to women who have not experienced such violence [5]. Across many countries, males are at even greater risk of severe non-fatal injuries as a result of interpersonal violence outside the home, compared to females, with studies of English populations finding that males are significantly more likely to be admitted to hospital or present at an emergency department for an assault-related injury [6–8]. Addressing the physical and mental health consequences of interpersonal violence imposes substantial burdens and costs on health services [9]. The estimated cost of non-fatal interpersonal violence amongst adults (aged 16+ years) to the NHS and other health care providers in England and Wales was over £1.8 billion during the year 2015/2016 [10]. Other cost estimates show that the costs of violence to the NHS are equivalent to those of other public health issues, such as alcohol and tobacco [11]. Thus, interpersonal violence has high individual, economic, and health service costs and represents an important public health issue. An improved understanding of the associated risk factors is crucial to informing effective intervention strategies to prevent, reduce, and respond to interpersonal violence.

Using a lifecourse perspective, there is evidence to support the concept of revictimisation that individuals who experience victimisation in childhood are at increased risk for subsequent victimisation in later life [12, 13]. Historically, much research on outcomes associated with child abuse have examined the impacts of individual types of abuse in isolation, with a review of studies in 2001 finding that only a small minority of published work in the area included more than one form of abuse [14]. Early studies of violence revictimisation thus tended to focus on a specific type of child abuse and its relationship with a particular, often similar, form of victimisation in adulthood. For example, studies have found that child sexual abuse increased the likelihood of being raped in adulthood by between two- and eleven-fold [15, 16], whilst exposure to domestic abuse as a child has been associated with being a victim of domestic abuse as an adult [17] and exposing their own children to parental conflict [18]. This body of research implied that types of abuse occur individually. However, more recent research has demonstrated that child abuse, and other types of adversity, typically co-occur [19–21] and consequently studies which attribute risk of revictimisation to one specific form of abuse may be misleading.

Over the past two decades, increasing attention has focused on the contribution of co-occurring adverse childhood experiences (ACEs; including physical, sexual, and emotional abuse as well as markers of household dysfunction such as domestic violence) to a host of poor outcomes in adulthood, including experiencing violence [20]. Crucially, a recent systematic review and meta-analyses examining ACEs and health during adulthood found that violence victimisation was amongst the outcomes most strongly associated with experiencing multiple types of ACEs, with pooled odds ratios indicating individuals with four or more ACEs were over seven times more likely to have been a victim of violence, compared to those reporting none [22]. Estimates from a national study of households in England showed that a history of exposure to ACEs could account for approximately half of all adults experiencing violence victimisation in the past year [19]. This equates annually to over one million individuals being assaulted at the national population level [19]. Thus, addressing abuse and adversity in childhood is an important public health issue which is likely to have a downstream effect on levels of violence victimisation amongst adults.

Crucially, these studies also demonstrate that childhood adversity operates in an additive manner whereby there is a positive relationship between exposure to higher numbers of ACEs and increased risk of a range of poor outcomes [19, 20, 22]. Findings from a national household survey in England demonstrated the graded relationship between the number of ACEs experienced and the risk of violence victimisation as an adult. Compared to those who had no history of ACEs, individuals with experience of one ACE were over one and a half times more likely to have been a victim of violence in the past year, increasing to over four times more likely for those with 2–3 ACEs, and over seven times more likely for those with four or more ACEs [19]. This dose-response relationship between childhood adversity and experience of violence in adulthood has also been replicated in several studies across different countries [23–26]. To date however, there are a limited number of studies which examine the relationship between childhood abuse and different forms of adult interpersonal violence. Findings from US studies of a health care population demonstrated a strong graded relationship between the number of ACEs experienced and risk of being a victim of intimate partner violence [27] and sexual violence in adulthood [28]. However, the generalisability of the findings is limited by the use of a non-representative health care sample.

Studies to date have typically focused on the cumulative burden of ACEs or focused specifically on an individual ACE. The cumulative ACE approach has provided a means of synthesising complex evidence to demonstrate the impact of adversity on a wide range of public health outcomes, and, in some countries, this has been instrumental in affecting national policy changes [29, 30]. Consistent findings across ACE studies, often despite differences in methodology, settings, and types of ACEs measured, suggest some consistency in the impact of ACEs in different populations [22]. However, it has also been highlighted that such aggregative approaches are best supplemented by complementary studies to investigate the potentially differential impact of different types, or combination of types, of adversity [20, 22, 31, 32]. One ACE study considered types of abuse in addition to the number of types on risk of sexual violence in adulthood [28]. Crucially, the study demonstrated that, whilst all ACE variables were significantly associated with sexual violence victimisation in adulthood, child sexual abuse was the strongest predictor of adult sexual violence. Furthermore, consistent with the ACE cumulative impact hypothesis, additional adversities experienced by child sexual abuse victims increased the risk of adulthood sexual violence, beyond the risk associated with child sexual abuse alone [28]. Findings from the study suggest both the type of abuse and the number of types are important. However, conclusions from the study were limited by the health care population sample and, crucially, the focus on one specific type of adulthood violence. There is evidence to suggest that different combinations of types may be associated with different outcomes in adulthood [33]. However, study of the specificity of abuse on different types of revictimisation has largely been neglected.

The primary aim of the current study is to examine the relationship between abuse in childhood and violence revictimisation in adulthood, including intimate partner violence, sexual violence and physical assault, using data from a nationally representative general population sample. Given that, to date, research has typically examined the impacts of childhood adversity either from an aggregative perspective, with less regard to type of adversity, or a type perspective, with less regard to multi-type victimisation, we include consideration of both type of childhood abuse and number of types in the current study.

## Methods

### Data source

Data for the current study was drawn from the 2015/2016 Crime Survey for England and Wales (CSEW) [34]. The CSEW is a face-to-face survey in which permanent residents of households in England and Wales are asked about their experiences of crime. It includes adults aged 16 and over except those living in institutions or group accommodation (e.g. halls of residence, care homes, homeless shelters). Face-to-face interviews are carried out using computer-assisted personal interviewing for the victimisation module, including all questions on interpersonal violence and household crime, and computer-assisted self-interviewing for self-completion modules on sensitive issues such as domestic abuse, sexual assault, and stalking [35]. The CSEW also occasionally includes modules of questions on topical issues, and the 2015/2016 CSEW incorporated, for the first time, a self-complete module of questions which asked adult respondents whether they experienced abuse as a child (before the age of 16 years).

The CSEW sample design and methods are discussed in detail elsewhere [35, 36]. The CSEW uses a random probability approach stratified by police force area and then by lower super output areas (LSOAs; geographical areas with a population average of 1500 individuals [37]). The survey is weighted to adjust for possible non-response bias to ensure the sample reflects the profile of the general population. Thus, the core sample is designed to be representative of the population of households in England and Wales. The Postcode Address File is used as the address source for the CSEW, with postcodes and LSOAs linked via the Office for National Statistics’ (ONS) National Statistics Postcode Lookup database, allowing addresses to be allocated to sample strata. Within each police force area, the number of addresses is based on the target number of interviews to be achieved across the year divided by the estimated address conversion rate. The number of addresses selected for the 2015/2016 CSEW varied within each sample unit but averaged 38, with 54,074 addresses initially sampled, of which 48,991 were eligible (i.e. occupied). Within each eligible household, one adult was randomly selected for interview based on a standard selection algorithm. A total of 35,146 interviews were conducted, accounting for a final response rate of 71.7% [36]. The self-complete module of the survey, which includes domestic abuse, sexual assault, and child abuse, is restricted to adults aged 16–59 years due to considerations around data quality and respondent burden [38]. Thus, the total sample size in the current study was 21,845.

### Measures

#### Childhood abuse

Childhood abuse was assessed using questions from the self-completion module: experience of abuse during childhood. Child physical abuse (CPA) consisted of one item indicating that respondents were deliberately pushed, held down, or slapped hard; kicked, bitten, or hit with a fist or something else; had something thrown at them; were choked or had someone attempt to strangle them; hit or attacked with a weapon or an object; burned; or had some other kind of force inflicted against them in a non-sexual way.^1^ Child psychological abuse (CPsychA) was measured with one item where the respondent indicates that they were not loved, told that they should never have been born, threatened with abandonment or eviction from family home, repeatedly belittled, physically threatened, or emotionally neglected. Child sexual abuse (CSA) was measured using three items which included sexual assault by rape or penetration, including attempts, or other sexual assaults including indecent exposure or sexual touching. Children witnessing domestic violence (CWDV)^2^ was measured using one item which asked if the respondent had witnessed domestic violence or abuse, including any psychological, physical, or sexual assault in the home, during childhood. All items pertained to abuse the respondent had experienced before the age of 16 years and which was perpetrated by an adult. The response categories were yes, no, do not know, and do not wish to answer. A count variable of the number of types of childhood abuse was created and categorised as none, single type, and multiple types.

#### Adulthood violence victimisation

Three types of adulthood violence victimisation were included in the current study: non-sexual intimate partner violence (IPV) since age 16 years, sexual violence (SV) since 16 years, and past year physical assault (PA). Nine items from the self-completion module: domestic violence, sexual victimisation, and stalking module, were combined to create one variable to indicate the presence of IPV (Additional file 1: Table A1). Eight items from the same module were combined to create one variable to indicate the presence of SV. Physical assault in the past 12 months was assessed using a derived variable (from the non-victim form CSEW 2015/2016 dataset), produced by the Home Office/ONS, which combined all CSEW offence codes^3^ related to assault, including violence with and without injury, including both completed and attempted incidents. As questions used to create the derived variable for physical assault are asked of all survey participants, but questions on intimate partner violence and sexual violence are limited to adults aged 16–59 years, the sample for the current study was limited to respondents aged 16–59 years to ensure the same sample of individuals was used for each violence outcome.

#### Covariates

Demographic characteristics controlled for in logistic regressions included sex (male or female), age (years 16–59), ethnicity (white or others), and deprivation. Deprivation is measured in the CSEW using the English Index of Multiple Deprivation (IMD) 2015 [39] and Welsh IMD 2014 [40], which comprise of different domains, or measures, of deprivation including, for example, income, employment, health, and crime. Measuring deprivation in this way allows the inclusion of a wide range of aspects of an individual’s living conditions [41]. The IMD is the official measure of relative deprivation for small geographies called lower super output areas (LSOAs; areas with mean population of 1500 [37]). LSOAs are categorised into deprivation quintiles (1, most deprived; 5, least deprived) based on their ranking in the IMD within their respective countries. Participants in the CSEW are then grouped based on the quintiles of IMD score in the country where they reside.

### Statistical analyses

All analyses were undertaken using SPSS (v24). Similar to practice elsewhere, missing values for childhood abuse measures were coded as a negative response [19, 42]. This approach allows the inclusion of cases where a data point is missing in one variable and not in another, controlling for the effects of missing data [42]. Only individuals with complete data for the violence outcome of interest (i.e. intimate partner violence, sexual violence or physical assault), age, sex, and ethnicity were included in the logistic regression analysis. Where individuals did not answer all relevant questions, final sample sizes are presented. Logistic regression was used to examine the independent relationships between child abuse types, and number of types, and adult violence outcomes, after controlling for sociodemographics. To examine the relationship between childhood abuse and adulthood violence victimisation, three regression models were ran for each violence outcome (each controlling for sociodemographics). Model 1 included child abuse count; model 2 included all individual types of child abuse; and model 3 included both child abuse count and all individual types of child abuse.

## Results

Information about the demographic characteristics of individuals included in the current study is provided in Additional file 1: Table A2. 18.6% (*n* = 4058) of adults aged 16–59 years reported at least one form of childhood abuse, with 10.6% (*n* = 2316) reporting a single type of child abuse and 8.0% (*n* = 1742) two or more types. Sample prevalence rates of individual types of childhood abuse are presented in Table 1. All child abuse types were significantly associated with each other, with the strongest association between CPsychA and CPA, and the weakest association between CSA and CWDV (Additional file 1: Table A3). Females were more likely than males to have experienced at least one type of child abuse and also more likely to report CPsychA, CSA, and CWDV (Table 1). There was also a significant association between each individual type of childhood abuse and age, and abuse count and age, with the youngest age group having the lowest prevalence across all CM categories (Table 1). White respondents were more likely to report all individual types of childhood abuse and multiple forms of abuse than individuals of other ethnicities (Table 1). The relationship between deprivation and prevalence of childhood abuse differed by type of abuse, with higher prevalence of CPsychA and CWDV amongst more deprived quintiles (1–3) compared to less deprived quintiles (4 and 5). The prevalence of CSA however was higher amongst less deprived quintiles (3–5) compared to more deprived quintiles (1 and 2). Childhood abuse count was also significantly associated with deprivation, with higher levels of deprivation (quintiles 1–3) associated with higher prevalence of multiple forms of childhood abuse (Table 1). Overall, differences between deprivation levels were relatively small, and abuse was prevalent across all quintiles of deprivation (Table 1).

**Table 1:** Bivariate relationships between sociodemographics and childhood abuse (< 16 years)

		Childhood psychological abuse	Childhood physical abuse	Childhood sexual abuse	Childhood witnessing domestic violence	Childhood abuse count		
						None	Single type	Multiple types
All	% (*N*)	9.0 (1971)	6.9 (1512)	6.9 (1513)	8.1 (1777)	81.4 (17,787)	10.6 (2316)	8.0 (1742)
Sex								
Male	% (*n*)	6.8 (678)	6.9 (680)	2.8 (275)	6.0 (597)	86.0 (8517)	8.4 (829)	5.7 (563)
Female	% (*n*)	10.8 (1293)	7.0 (832)	10.4 (1238)	9.9 (1180)	77.7 (9270)	12.5 (1487)	9.9 (1179)
*χ*^2^		104.547	0.082	483.524	107.503	250.723		
*p*		< 0.001	0.774	< 0.001	< 0.001	< 0.001		
Age (years)								
16–19	% (*n*)	7.8 (72)	3.6 (33)	2.1 (19)	4.1 (38)	88.0 (809)	7.9 (73)	4.0 (37)
20–29	% (*n*)	9.8 (381)	6.5 (254)	4.8 (187)	8.9 (345)	82.5 (3210)	9.5 (370)	8.0 (310)
30–39	% (*n*)	8.4 (450)	7.0 (374)	5.7 (304)	8.1 (437)	82.4 (4427)	10.2 (548)	7.4 (399)
40–49	% (*n*)	9.8 (587)	7.4 (439)	8.2 (492)	8.9 (532)	79.9 (4764)	11.2 (667)	9.0 (534)
50–59	% (*n*)	8.4 (481)	7.2 (412)	9.0 (511)	7.5 (425)	80.3 (4577)	11.5 (658)	8.1 (462)
*χ*^2^		14.355	19.393	127.254	30.856	54.025		
*p*		< 0.01	< 0.01	< 0.001	< 0.001	< 0.001		
Deprivation quintile								
1 (most deprived)	% (*n*)	9.6 (433)	7.2 (322)	6.4 (287)	8.4 (379)	82.2 (3700)	9.3 (420)	8.4 (379)
2	% (*n*)	9.4 (414)	7.8 (342)	6.0 (264)	9.2 (407)	80.9 (3562)	10.3 (454)	8.8 (386)
3	% (*n*)	9.7 (419)	6.9 (297)	7.5 (321)	8.3 (355)	80.6 (3466)	11.0 (473)	8.4 (361)
4	% (*n*)	8.7 (377)	6.4 (277)	7.6 (331)	7.7 (336)	81.3 (3543)	11.1 (485)	7.6 (330)
5 (least deprived)	% (*n*)	7.7 (328)	6.4 (274)	7.2 (310)	7.0 (300)	82.0 (3516)	11.3 (484)	6.7 (286)
*χ*^2^		16.027	9.320	13.569	16.304	27.499		
*p*		< 0.01	0.054	< 0.01	< 0.01	< 0.01		
Ethnicity								
White	% (*n*)	9.5 (1813)	7.2 (1380)	7.3 (1395)	8.5 (1613)	80.4 (15,317)	11.2 (2129)	8.4 (1598)
Others	% (*n*)	5.7 (157)	4.7 (131)	4.3 (118)	5.9 (164)	88.1 (2436)	6.8 (187)	5.2 (143)
*χ*^2^		42.967	23.216	34.536	20.495	93.120		
*p*		< 0.001	< 0.001	< 0.001	< 0.001	< 0.001		

Note. Limited to adults aged 16–59 years who completed the CSEW 2015/2016 self-completion modules

### Intimate partner violence (IPV)

Of the sample, 18.9% (*n* = 3681) had experienced some form of intimate partner violence (IPV) since the age of 16. In bivariate analyses, all individual types of childhood abuse and the combined variable childhood abuse count were significantly associated with IPV (Additional file 1: Table A4). Each individual type of childhood abuse remained significantly associated with IPV, after controlling for sociodemographics (age, gender, ethnicity, and deprivation), with adjusted odds ratios (AORs) ranging from 3.21 (CSA) to 5.09 (CPsychA) (Additional file 1: Table A4). After controlling for sociodemographics, child abuse count indicated a graded relationship with IPV, with the AOR for experiencing multiple types of abuse approximately twice as high as the AOR for a single form of abuse (Table 2; model 1). When all individual types of childhood abuse were entered simultaneously into the model, relationships with IPV were attenuated (compared to unadjusted measures) but all individual types remained independently associated with increased odds of IPV (Table 2; model 2). When childhood abuse count was added into the model along with all individual types, abuse count and CPsychA and CPA independently predicted IPV (Table 2; model 3).

**Table 2:** Associations between childhood abuse (< 16 years) and lifetime intimate partner violence victimisation (≥ 16 years) (*N* = 19,471)

		Model 1				Model 2				Model 3			
		Sig.	AOR	95% CIs		Sig.	AOR	95% CIs		Sig.	AOR	95% CIs	
				LL	UL			LL	UL			LL	UL
Childhood psychological abuse		–				< 0.001	2.78	2.45	3.15	< 0.001	1.56	1.33	1.82
Childhood physical abuse		–				< 0.001	1.79	1.55	2.07	< 0.05	1.21	1.03	1.42
Childhood sexual abuse		–				< 0.001	2.06	1.83	2.33	*NS*			
Childhood witnessing domestic violence		–				< 0.001	1.76	1.55	2.00	*NS*			
Childhood abuse count	None (ref.)	< 0.001				–				< 0.001			
	Single type	< 0.001	3.27	2.95	3.62	–				< 0.001	2.85	2.54	3.19
	Multiple types	< 0.001	6.41	5.75	7.15	–				< 0.001	3.96	3.22	4.87
Sex	Female	< 0.001	2.52	2.32	2.74	< 0.001	2.53	2.33	2.76	< 0.001	2.57	2.36	2.79
Age	50–59 (ref.)					< 0.001				< 0.001			
	16–19	< 0.01	0.65	0.49	0.85	< 0.05	0.64	0.48	0.83	< 0.01	0.62	0.48	0.82
	20–29	*NS*	0.97	0.86	1.10	*NS*	0.96	0.85	1.08	*NS*	0.96	0.85	1.09
	30–39	< 0.05	1.14	1.02	1.27	< 0.05	1.13	1.02	1.26	< 0.05	1.13	1.02	1.26
	40–49	< 0.001	1.25	1.13	1.39	< 0.001	1.24	1.12	1.37	< 0.001	1.25	1.12	1.38
Ethnicity	White	< 0.001	1.91	1.65	2.21	< 0.001	1.97	1.70	2.28	< 0.001	1.91	1.65	2.21
Deprivation	Least deprived (ref.)					< 0.001				< 0.001			
	Most deprived—1	< 0.001	1.43	1.26	1.62	< 0.001	1.40	1.23	1.58	< 0.001	1.42	1.25	1.61
	2	< 0.001	1.40	1.23	1.58	< 0.001	1.40	1.24	1.58	< 0.001	1.39	1.23	1.58
	3	< 0.001	1.26	1.12	1.43	< 0.001	1.25	1.11	1.42	< 0.001	1.26	1.11	1.42
	4	*NS*	1.11	0.98	1.25	*NS*	1.10	0.97	1.25	*NS*	1.11	0.98	1.25

Notes. Limited to adults aged 16–59 years who completed the CSEW 2015/2016 self-completion modules. *AOR* adjusted odds ratio, *95% CIs* 95% confidence intervals, *LL* lower limit, *UL* upper limit

Model 1: Childhood abuse count was entered in a regression, controlling for sociodemographics (sex, age, ethnicity, and deprivation quintiles)

Model 2: All childhood abuse types were entered in a regression, controlling for sociodemographics

Model 3: All childhood abuse types and childhood abuse count were entered into a regression, controlling for sociodemographics

### Sexual violence (SV)

Of the sample, 13.0% (*n* = 2623) had experienced sexual violence (SV) since age 16. In unadjusted analyses, all individual types of childhood abuse and childhood abuse count were significantly associated with SV (Additional file 1: Table A4). After controlling for sociodemographics (age, gender, ethnicity, and deprivation), each individual type of childhood abuse remained significantly associated with SV, with AORs ranging from 3.40 (CWDV) to 5.80 (CSA) (Additional file 1: Table A4). Childhood abuse count also remained significantly associated with SV, after controlling for sociodemographics and indicated a graded relationship (Table 3; model 1). When all individual types of childhood abuse were entered simultaneously into the same model, relationships were weakened but remained significantly associated with increased odds of SV (Table 3; model 2). When childhood abuse count was added into the model along with all individual types, abuse count and CPsychA and CSA independently predicted SV (Table 3; model 3).

**Table 3:** Associations between childhood abuse (< 16 years) and lifetime sexual violence victimisation (≥ 16 years) (*N* = 20,129)

		Model 1				Model 2				Model 3			
		Sig.	AOR	95% CIs		Sig.	AOR	95% CIs		Sig.	AOR	95% CIs	
				LL	UL			LL	UL			LL	UL
Childhood psychological abuse		–				< 0.001	2.53	2.19	2.92	< 0.001	1.63	1.34	1.98
Childhood physical abuse		–				< 0.001	1.50	1.27	1.78	*NS*			
Childhood sexual abuse		–				< 0.001	4.20	3.71	4.75	< 0.001	2.47	2.10	2.91
Childhood witnessing domestic violence		–				< 0.001	1.46	1.26	1.69	*NS*			
Childhood abuse count	None (ref.)	< 0.001				–				< 0.001			
	Single type	< 0.001	3.58	3.195	4.01	–				< 0.001	2.16	1.85	2.52
	Multiple types	< 0.001	6.89	6.11	7.76	–				< 0.001	3.06	2.42	3.86
Sex	Female	< 0.001	6.77	5.99	7.66	< 0.001	6.36	5.62	7.21	< 0.001	6.29	5.56	7.12
Age	50–59 (ref.)	*NS*				*NS*				*NS*			
	16–19												
	20–29												
	30–39												
	40–49												
Ethnicity	White	< 0.001	1.51	1.28	1.78	< 0.001	1.55	1.32	1.84	< 0.001	1.52	1.28	1.79
Deprivation	Least deprived (ref.)	< 0.001				< 0.001				< 0.001			
	Most deprived—1	< 0.001	0.72	0.62	0.83	< 0.001	0.71	0.61	0.82	< 0.001	0.72	0.62	0.84
	2	*NS*	0.88	0.76	1.01	*NS*	0.91	0.79	1.05	*NS*	0.91	0.79	1.05
	3	*NS*	0.97	0.85	1.12	*NS*	0.98	0.85	1.12	*NS*	0.98	0.85	1.12
	4	*NS*	1.14	0.99	1.30	*NS*	1.14	0.99	1.30	*NS*	1.14	0.99	1.31

Notes. Limited to adults aged 16–59 years who completed the CSEW 2015/2016 self-completion modules. *AOR* adjusted odds ratio, *95% CIs* 95% confidence intervals, *LL* lower limit, *UL* upper limit

Model 1: Childhood abuse count was entered in a regression, controlling for sociodemographics (sex, age, ethnicity, and deprivation quintiles)

Model 2: All childhood abuse types were entered in a regression, controlling for sociodemographics

Model 3: All childhood abuse types and childhood abuse count were entered in a regression, controlling for sociodemographics

### Physical assault (PA)

Of the sample, 2.4% (*n* = 531) had experienced a physical assault (PA) in the past 12 months. In unadjusted analyses, all individual types of childhood abuse and childhood abuse count were significantly associated with PA (Additional file 1: Table A4). After controlling for sociodemographics (age, gender, ethnicity, and deprivation), each individual type of childhood abuse remained significantly associated with PA, with AORs ranging from 2.41 (CSA) to 3.14 (CPsychA) (Additional file 1: Table A4). Childhood abuse count also remained significantly associated with PA and indicated a graded relationship (Table 4; model 1). When all types of childhood abuse were entered simultaneously into the same model, CPA was no longer significantly associated with PA. Relationships between CPsychA, CSA, and CWDV, and PA were attenuated but remained statistically significant (Table 4; model 2). When childhood abuse count was added into the model along with all individual types, abuse count and CPsychA independently predicted PA (Table 4; model 3).

**Table 4:** Associations between childhood abuse (< 16 years) and past year physical assault victimisation (≥ 16 years) (*N* = 21,810)

		Model 1				Model 2				Model 3			
		Sig.	AOR	95% CIs		Sig.	AOR	95% CIs		Sig.	AOR	95% CIs	
				LL	UL			LL	UL			LL	UL
Childhood psychological abuse		–				< 0.001	2.37	1.84	3.06	< 0.01	1.61	1.13	2.31
Childhood physical abuse		–				*NS*				*NS*			
Childhood sexual abuse		–				< 0.01	1.61	1.21	2.14	*NS*			
Childhood witnessing domestic violence		–				< 0.01	1.50	1.14	1.98	*NS*			
Childhood abuse count	None (ref.)	< 0.001				–				< 0.001			
	Single type	< 0.001	2.16	1.70	2.75	–				< 0.001	1.86	1.42	2.45
	Multiple types	< 0.001	3.37	2.68	4.24	–				< 0.001	2.25	1.53	3.32
Sex	Female	< 0.001	0.68	0.57	0.81	< 0.001	0.67	0.56	0.80	< 0.001	0.69	0.58	0.82
Age	50–59 (ref.)	< 0.001				< 0.001				< 0.001			
	16–19	< 0.001	2.13	1.40	3.27	< 0.01	2.08	1.36	3.19	< 0.01	2.07	1.35	3.17
	20–29	< 0.001	2.20	1.68	2.88	< 0.001	2.16	1.65	2.83	< 0.001	2.17	1.66	2.83
	30–39	< 0.01	1.55	1.19	2.03	< 0.01	1.55	1.18	2.03	< 0.01	1.54	1.18	2.02
	40–49	< 0.05	1.37	1.05	1.80	< 0.05	1.35	1.03	1.76	< 0.05	1.36	1.04	1.78
Ethnicity	White	< 0.01	1.72	1.26	2.36	< 0.001	1.75	1.28	2.39	< 0.01	1.72	1.26	2.35
Deprivation	Least deprived (ref.)	< 0.001				< 0.001				< 0.001			
	Most deprived—1	< 0.001	1.92	1.44	2.55	< 0.001	1.88	1.41	2.50	< 0.001	1.91	1.43	2.54
	2	< 0.01	1.52	1.13	2.04	< 0.01	1.52	1.13	2.05	< 0.01	1.52	1.13	2.04
	3	*NS*	1.13	0.82	1.54	*NS*	1.117	0.82	1.53	*NS*	1.12	0.82	1.54
	4	*NS*	1.26	0.93	1.71	*NS*	1.251	0.92	1.70	*NS*	1.26	0.93	1.71

Notes. Limited to adults aged 16–59 years who completed the CSEW 2015/2016 self-completion modules. *AOR* adjusted odds ratio, *95% CIs* 95% confidence intervals, *LL* lower limit, *UL* upper limit

Model 1: Childhood abuse count was entered in a regression, controlling for sociodemographics (sex, age, ethnicity, and deprivation quintiles)

Model 2: All childhood abuse types were entered in a regression, controlling for sociodemographics

Model 3: All childhood abuse types and childhood abuse count were entered in a regression, controlling for sociodemographics

## Discussion

Using data from a nationally representative sample of adults resident in England and Wales, findings from the study demonstrated strong associations between childhood abuse and three types of violence revictimisation in adulthood: past year physical assault (PA), and intimate partner violence (IPV) and sexual violence victimisation (SV) since age 16 years. Consistent with early approaches in the field of child abuse research [15–18], examining types of abuse individually in the current study demonstrated that all types were significantly associated with each adult violence outcome, after controlling for sociodemographics. Crucially, and consistent with more recent research [19], our findings also showed correlation and co-occurrence of abuse types, providing further evidence that multiple types of abuse should be considered together in both research and practice. Thus, studies which examine a single form of abuse could erroneously attribute the increased risk of revictimisation as the unique impact of the type of abuse under study. When all individual types of abuse were considered together in the current study, most relationships with adult violence outcomes were attenuated but remained significant, suggesting child abuse in general, regardless of type, increases the risk of PA, IPV, and SV in adulthood. Further, consistent with findings elsewhere [19, 22, 23, 26–28], the current study demonstrated a cumulative impact of child abuse on risk of violence revictimisation. Compared to individuals who experienced no abuse in childhood, those who experienced one form of abuse were over twice as likely to report PA in the past year and three times as likely to have experienced IPV and/or SV since age 16 years. Crucially, individuals who experienced multiple types were three, six, and seven times more likely than those who experienced no abuse to have experienced PA, IPV, and SV, respectively. Thus, whilst most abuse types were independently related to past year PA and IPV and SV since age 16 years, the strongest predictor of each adulthood violence outcome was experiencing multiple forms of abuse in childhood.

Whilst child abuse in general, regardless of type, was associated with increased risk of revictimisation particularly for individuals who had experienced multiple types, the current study expanded previous research on revictimisation by demonstrating that when the effects of experiencing multiple forms of abuse were accounted for, type of abuse remained predictive of adult violence outcomes. Such findings are consistent with previous research showing that specific types of adversity account for impairment in areas such as mental health above the cumulative impact of number of types of abuse [43, 44]. This suggests that the effects of particular combinations of childhood abuse may be obscured when each type is given an equal weight, as in the aggregative count approach. Crucially, the type or combination of types which increased risk differed by violence outcome in the current study. After accounting for sociodemographics and multi-type victimisation, CPsychA and CPA remained significantly associated with IPV; CPsychA and CSA remained associated with SV; and CPsychA was the only childhood abuse type which remained associated with PA. That differential combinations of abuse are associated with different violence outcomes is in line with previous studies that consider both type and number of types, and which found specificity of types of abuse on mental illness outcomes in child and adult samples [33, 45].

Childhood abuse is not a direct causal factor of violence victimisation in adulthood but rather abuse is likely to disrupt normative developmental processes, such as secure attachment to the caregiver which enables children to regulate behaviour and emotions [46, 47], create difficulties relating to others and forming healthy relationships [48–50], and cause problems in areas such as aggression, hyper arousal, and impulse control [51–53]. These factors may function as adaptive reactions in abusive environments, but conversely may also increase risk of revictimisation. For example, experiencing hyper arousal on a consistent basis may increase vigilance to danger cues in the environment but equally reduce the ability to differentiate serious threat cues from less serious, increasing risk of revictimisation [54]. Aggression and problems with impulse control are associated with increased likelihood to associate with delinquent peers and involvement in violence [55–57], whilst some research suggests experiencing or witnessing violent behaviour as a child might lead to learned expectations that the use of violence to resolve conflicts in relationships is normative or that complying with a violent partner is a means of reducing further harm [58, 59]. Some studies suggest that pathways may differ by adulthood violence outcome, with evidence that the association between CSA and SV is mediated via problematic alcohol use and risky sexual behaviour including early onset sexual activity, multiple sexual partners, and promiscuous sexual behaviour [60, 61], whilst the association between childhood adversity and IPV has been found to be mediated by psychosocial characteristics including depression, anxiety, and impulsivity [62]. Although further research is required, such pathways and mediating processes between child abuse and adulthood revictimisation may represent important targets for public health interventions [63]. However, universal and selective interventions which promote positive parent-child relationships and supportive relationships from other appropriate adults have the potential to prevent and/or mitigate the impact of childhood abuse [64] and are likely to be the most effective at the population level in reducing multiple forms of revictimisation in adulthood. The health sector plays a key role in implementing such intervention strategies, with the Safe Environment for Every Kid (SEEK) model in paediatric primary care and the Nurse-Family Partnership home visitation programme, examples of two programmes implemented in health care settings or by health care personnel, which are effective in reducing child abuse and domestic violence and improving child and parental health outcomes [65–67]. Findings from our study suggest such programmes may also indirectly reduce risk of violence victimisation across the lifecourse, thereby reducing the burden and cost associated with adulthood violence victimisation to health care systems.

That child abuse is associated with repeat victimisation in adulthood also has important implications for adult health service provision as, similar to the additive manner in which child abuse impacts health and wellbeing, revictimisation in adulthood can further compound and exacerbate the effects of child abuse [68, 69]. Further, the burden of interpersonal violence is disproportionately borne by small numbers of adults who experience high frequencies of victimisation, particularly in the case of IPV [70–72]. Thus, whilst not accounted for in the current study, such individuals are likely to be repeated victims of violence of one or more types in adulthood, further compounding trauma. Individuals who experience both child abuse and violence as an adult are at increased risk of trauma-associated health issues including mental health problems [73–75], substance use disorders [76], and adverse pregnancy outcomes [77]. Critically, many of the health impacts associated with interpersonal violence also represent adversities for the next generation, perpetuating the intergenerational transmission of adversity. Multi-sector efforts are increasingly underway to develop trauma-informed services, which recognise the relationship between current health and social problems and previous experience of trauma [78, 79]. Health sector services, such as substance misuse services, sexual health clinics, and mental health services, are likely to encounter higher proportions of individuals presenting with chronic adversity in their childhood histories compared to the general population [42, 80], and by adopting a trauma-informed approach to treatment, can better understand and address patient issues [81, 82]. Thus, the health sector can play a crucial role in interrupting the cycle of violence between childhood and adulthood victimisation, through both primary prevention of child abuse and trauma-informed approaches to addressing subsequent health and social outcomes and concurrently prevent or reduce levels of adversity in successive generations. Such efforts represent a lifecourse approach towards violence prevention and a focus on the early drivers of poor health and social outcomes. The UN 2030 Agenda for Sustainable Development Goals (SDGs) provides the political endorsement and multi-sectoral framework for such an approach, and whilst several of the SDGs directly concern violence, the achievement of other SDGs (e.g. good health and wellbeing, reduced inequalities) is also dependent on efforts across all sectors to address violence and its risk factors [83, 84].

### Limitations

In light of the findings, several limitations of the study should also be considered. As with many population surveys, the CSEW is cross-sectional in design; thus, causality in the current study cannot be established. However, the temporal ordering of child abuse predictors and adult violence outcomes suggests child abuse increases risk of revictimisation. The use of 16 years by the CSEW as the cut-off between childhood and adulthood may mean that under other definitions of childhood abuse (i.e. before 18 years) some experiences of revictimisation in adulthood in the current study would be considered other incidences of child abuse. Further, questions on child abuse in the CSEW are not from a standardised instrument for measuring child abuse, although they are similar in format and content to questions used in adverse childhood experiences literature [19, 20]. Here we aimed to examine the strength of associations between exposure to child abuse and subsequent experiences in adulthood of physical and sexual assault. For those purposes, the use of logistic regression techniques is consistent with studies elsewhere [19, 20]. Further analyses using statistical mediation techniques could allow additional understanding of the links between child abuse and adult outcomes in future studies. Data on behavioural and individual-level (beyond sociodemographics) factors, which may be potential mechanisms for the association between child abuse and risk of revictimisation, were unavailable in the CSEW data set to consider for inclusion in the analyses. Behavioural factors, such as perpetration of violence, delinquency, and substance use, and individual-level factors, such as self-regulation and psychopathology, have previously been demonstrated to be associated to child abuse and/or adulthood violence victimisation [85–87]; thus, investigation of the mechanisms through which revictimisation occurs is an important area for future research. The retrospective nature of the survey necessitates participants recalling abuse in childhood. Studies have shown complex trauma in childhood can be associated with repression of memory and dissociation, thus potentially increasing risk of underreporting of abuse [88]. The CSEW is a population household survey, thus excludes higher risk populations such as patients in psychiatric facilities, prisoners, or individuals experiencing homelessness where rates of child abuse and adulthood violence victimisation are higher than the general population [89–91]. Markers of household dysfunction have been demonstrated in other studies to further increase the cumulative risk of adverse outcomes, including experiencing violence, associated with childhood abuse [28]. No information was available on other types of adversity; however, child abuse has been previously found to be one of the strongest predictor of adverse outcomes [28, 42, 92]. The 2015/2016 CSEW did not however include all forms of child abuse, and although emotional neglect is covered within the definition of psychological abuse, physical neglect in childhood is omitted. Further, there was insufficient information on other factors which have been demonstrated to impact later outcomes including age of onset, severity, and chronicity of abuse to allow inclusion in the current study [90, 93].

## Conclusions

Interpersonal violence, including both childhood and adulthood victimisation, is one of the most preventable causes of premature morbidity and mortality and represents key targets of the United Nation’s Sustainable Development Goals [83]. Whilst prevention of all forms of child abuse is an important goal for ethical, health, and economic reasons, evidence from the current study suggests such prevention efforts will also have a downstream effect on preventing interpersonal violence across the lifecourse, disrupting cycles of victimisation. Results here demonstrate that experiencing multiple types of abuse is the strongest risk factor for violence revictimisation and suggest that individual types of abuse may be differentially associated with different violence outcomes in adulthood when multi-type victimisation is accounted for. This suggests potentially different pathways between childhood abuse and different types of adulthood interpersonal violence, which may have important implications for intervention efforts targeted at mediating factors. With adulthood victimisation likely to compound the already detrimental effects of childhood abuse [68, 69], there is a compelling case for focusing global public health and health sector efforts on preventing and mitigating the impacts of childhood abuse. Such efforts will not only reduce risk of revictimisation across the lifecourse for individuals, but given that exposure to such violence also represents adversities for the next generation, it represents a crucial means of disrupting intergenerational cycles of violence.

## Supplementary information

**Additional file 1: Table A1.** Adulthood violence victimisation items. **Table A2.** Respondent sociodemographics. **Table A3.** Changes in odds of reporting any type of childhood abuse with experiencing any other childhood abuse. **Table A4.** Bivariate and adjusted associations between child abuse (< 16 years) and adult violence victimisation (≥16 years), CSEW 15/16 adults aged 16–59 years.

## Data Availability

The data that support the findings of this study are available from UK Data Service but restrictions apply to the availability of these data, which were used under licence for the current study, and so are not publicly available. Data are however available from the UK Data Service and with permission of the Office for National Statistics.

## Abbreviations

AORs: Adjusted odds ratios
ACEs: Adverse childhood experiences
CPA: Childhood physical abuse
CPsychA: Childhood psychological abuse
CSA: Childhood sexual abuse
CWDV: Childhood witnessing domestic violence
IMD: Index of Multiple Deprivation
IPV: Intimate partner violence
LSOAs: Lower super output areas
ONS: Office for National Statistics
PA: Physical assault
SV: Sexual violence

## Footnotes

1 Includes smacking or corporal punishment at school, but excludes peer on peer bullying.

2 Whilst we acknowledge children ‘experience’ domestic violence, we refer to ‘witnessing’ domestic violence to maintain consistency with the CSEW survey question.

3 Based on information collected from the victimisation modules, specially trained coders determine whether what is reported constitutes a crime, and if so, what offence code should be assigned to the crime.
