# Supplementary material for: Cycles of violence in England and Wales: the contribution of childhood abuse to risk of violence revictimisation in adulthood
Source: BMC Med. 2020 Nov 16;18:325. doi: 10.1186/s12916-020-01788-3 (PMC7667802; doi:10.1186/s12916-020-01788-3)
Supplement: Supplementary file 1 — Additional file 1: Table A1. Adulthood violence victimisation items. Table A2. Respondent sociodemographics. Table A3. Changes in odds of reporting any type of childhood abuse with experiencing any other childhood abuse. Table A4. Bivariate and adjusted associations between child abuse (< 16 years) and adult violence victimisation (≥16 years), CSEW 15/16 adults aged 16–59 years. [file 12916_2020_1788_MOESM1_ESM.docx]

**Table A1: Adulthood violence victimisation items**

| Adulthood violence victimisation variables | CSEW survey items | Response |
| --- | --- | --- |
| Intimate partner violence since age 16 years | **Since you were age 16 has a partner or ex-partner (by partner we mean a boyfriend, girlfriend, husband, wife or civil partner) ever done any of the following:** |  |
|  | Prevented you from having your fair share of the household money, stopped you from seeing friends and relatives, repeatedly belittled you to the extent that you felt worthless | Yes to any of the items |
|  | Frightened or threatened you in any way |  |
|  | Used force on you |  |
|  | Sent you more than one unwanted letter, text message or card that was either obscene or threatening and which caused you fear, alarm or distress? |  |
|  | Made more than one obscene, threatening nuisance or silent phone call to you which caused you fear, alarm or distress? |  |
|  | Waited or loitered outside your home or workplace on more than one occasion in a manner which caused you fear, alarm or distress? |  |
|  | Followed you around and watched you on more than one occasion in a manner which caused you fear, alarm or distress? |  |
|  | Sent you more than one unwanted email or social network message that was obscene or threatening and which caused you fear, alarm or distress? |  |
|  | Put personal, obscene or threatening information about you on the internet on more than one occasion and which caused you fear, alarm or distress? |  |
| Sexual violence since age 16 years | **Since you were 16 has anyone (partner, family member, friend or work colleague, someone you knew casually, or a stranger) ever:** |  |
|  | Ever indecently exposed themselves to you (i.e. flashing) in a way that caused you fear, alarm or distress | Yes to any of the items |
|  | Ever touched you in a sexual way (e.g. touching, grabbing, kissing or fondling) when you did not want it |  |
|  | Ever penetrated your [mouth, vagina or anus/mouth or anus] with their penis when you made it clear that you did not agree or when you were not capable of consent |  |
|  | Ever penetrated your [vagina or anus/anus] with an object (including their fingers) when you made it clear that you did not agree or when you were not capable of consent? |  |
|  | Ever attempted to penetrate your [mouth, vagina or anus/mouth or anus] with their penis when you made it clear that you did not agree or when you were not capable of consent |  |
|  | Ever attempted to penetrate your [vagina or anus/anus] with an object (including their fingers) when you made it clear that you did not agree or when you were not capable of consent? |  |
|  | Ever forced you to penetrate another person’s mouth, vagina or anus with your penis when you made it clear that you did not agree or when you were not capable of consent |  |
|  | Ever forced you to penetrate another person’s vagina or anus with an object (including your fingers) when you made it clear that you did not agree or when you were not capable of consent? |  |

**Table A2: Respondent sociodemographics**

|  | n | % |
| --- | --- | --- |
| Age (years) |  |  |
| 16-19 | 919 | 4.2 |
| 20-29 | 3890 | 17.8 |
| 30-39 | 5374 | 24.6 |
| 40-49 | 5965 | 27.3 |
| 50-59 | 5697 | 26.1 |
| Sex |  |  |
| Male | 9909 | 45.4 |
| Female | 11936 | 54.6 |
| Ethnicity |  |  |
| White | 19044 | 87.3 |
| Other | 2766 | 12.7 |
| Deprivation quintile |  |  |
| 1 (most deprived) | 4499 | 20.6 |
| 2 | 4402 | 20.2 |
| 3 | 4300 | 19.7 |
| 4 | 4358 | 19.9 |
| 5 (least deprived) | 4286 | 19.6 |

**Note.** Limited to adults aged 16-59 years who completed the CSEW 2015/16 self-completion modules.

**Table A3: Changes in odds of reporting any type of childhood abuse with experiencing any other childhood abuse**

|  |  |  | **95% CIs** | |
| --- | --- | --- | --- | --- |
|  | **Sig.** | **OR** | **LL** | **UL** |
| CPsychA*CPA | p<0.001 | 29.73 | 26.34 | 33.55 |
| CPsychA*CSA | p<0.001 | 6.38 | 5.66 | 7.19 |
| CPsychA*CWDV | p<0.001 | 18.44 | 16.49 | 20.62 |
| CPA*CSA | p<0.001 | 5.89 | 5.18 | 6.71 |
| CPA*CWDV | p<0.001 | 16.22 | 14.41 | 18.36 |
| CSA*CWDV | p<0.001 | 5.87 | 5.19 | 6.65 |

**Notes.** Limited to adults aged 16-59 years who completed the CSEW 2015/16 self-completion modules. OR, odds ratio; 95% CIs, 95% confidence intervals; LL, lower limit; UL, upper limit. CPsychA, childhood psychological abuse; CPA, childhood physical abuse; CSA, childhood sexual abuse; CWDV, childhood witnessing domestic violence.

**Table A4: Bivariate and adjusted associations between child abuse (<16 years) and adult violence victimisation (≥16 years), CSEW 15/16 adults aged 16-59 years.**

|  |  | % (n) | Sig. | AOR | 95% CIs | |
| --- | --- | --- | --- | --- | --- | --- |
|  |  |  |  |  | **LL** | **UL** |
| Intimate partner violence since age 16 | | | | | | |
| Childhood psychological abuse | Yes | 49.9 (953) | <0.001 | 5.09 | 4.60 | 5.63 |
|  | No | 15.5 (2728) |  |  |  |  |
| Childhood physical abuse | Yes | 46.4 (688) | <0.001 | 4.59 | 4.09 | 5.14 |
|  | No | 16.6 (2993) |  |  |  |  |
| Childhood sexual abuse | Yes | 44.6 (665) | <0.001 | 3.21 | 2.87 | 3.60 |
|  | No | 16.8 (3016) |  |  |  |  |
| Childhood witnessing domestic violence | Yes | 45.0 (781) | <0.001 | 3.85 | 3.46 | 4.28 |
|  | No | 16.3 (2900) |  |  |  |  |
| Childhood abuse count | None | 13.0 (2024) | <0.001 | ref. |  |  |
|  | Single type | 34.7 (780) | <0.001 | 3.27 | 2.95 | 3.62 |
|  | Multiple types | 51.4 (877) | <0.001 | 6.41 | 5.75 | 7.15 |
| Sexual violence since age 16 | | | | | | |
| Childhood psychological abuse | Yes | 35.9 (699) | <0.001 | 4.56 | 4.08 | 5.10 |
|  | No | 10.6 (1924) |  |  |  |  |
| Childhood physical abuse | Yes | 31.5 (471) | <0.001 | 4.15 | 3.64 | 4.72 |
|  | No | 11.5 (2152) |  |  |  |  |
| Childhood sexual abuse | Yes | 47.1 (707) | <0.001 | 5.80 | 5.16 | 6.52 |
|  | No | 10.3 (1916) |  |  |  |  |
| Childhood witnessing domestic violence | Yes | 31.7 (557) | <0.001 | 3.40 | 3.03 | 3.83 |
|  | No | 11.2 (2066) |  |  |  |  |
| Childhood abuse count | None | 8.3 (1334) | <0.001 | ref. |  |  |
|  | Single type | 26.3 (606) | <0.001 | 3.58 | 3.20 | 4.01 |
|  | Multiple types | 39.7 (683) | <0.001 | 6.89 | 6.11 | 7.76 |
| Physical assault past 12 months | | | | | | |
| Childhood psychological abuse | Yes | 6.2 (122) | <0.001 | 3.14 | 2.54 | 3.87 |
|  | No | 2.1 (409) |  |  |  |  |
| Childhood physical abuse | Yes | 5.4 (82) | <0.001 | 2.51 | 1.97 | 3.20 |
|  | No | 2.2 (449) |  |  |  |  |
| Childhood sexual abuse | Yes | 4.5 (68) | <0.001 | 2.41 | 1.84 | 3.16 |
|  | No | 2.3 (463) |  |  |  |  |
| Childhood witnessing domestic violence | Yes | 5.4 (96) | <0.001 | 2.60 | 2.06 | 3.27 |
|  | No | 2.2 (435) |  |  |  |  |
| Childhood abuse count | None | 1.9 (340) | <0.001 | ref. |  |  |
|  | Single type | 3.8 (88) | <0.001 | 2.16 | 1.70 | 2.75 |
|  | Multiple types | 5.9 (103) | <0.001 | 3.37 | 2.68 | 4.24 |

**Note.** AOR, adjusted odds ratio (adjusted for sex, age, ethnicity, and deprivation); 95% CIs, 95% confidence intervals; LL, lower limit; UL, upper limit.
